# Readability of patient education materials in ophthalmology: a single-institution study and systematic review

**DOI:** 10.1186/s12886-016-0315-0

**Published:** 2016-08-03

**Authors:** Andrew M. Williams, Kelly W. Muir, Jullia A. Rosdahl

**Affiliations:** 1 Michigan State University College of Human Medicine, Grand Rapids, MI USA; 2 Department of Ophthalmology, University of Pittsburgh Medical Center, Pittsburgh, PA USA; 3 Duke University Department of Ophthalmology, Durham, NC USA; 4 Durham VA Medical Center, Health Services Research and Development, Durham, NC USA

## Abstract

**Background:**

Patient education materials should be written at a level that is understandable for patients with low health literacy. The aims of this study are (1) to review the literature on readability of ophthalmic patient education materials and (2) to evaluate and revise our institution’s patient education materials about glaucoma using evidence-based guidelines on writing for patients with low health literacy.

**Methods:**

A systematic search was conducted on the PubMed/MEDLINE database for studies that have evaluated readability level of ophthalmic patient education materials, and the reported readability scores were assessed. Additionally, we collected evidence-based guidelines for writing easy-to-read patient education materials, and these recommendations were applied to revise 12 patient education handouts on various glaucoma topics at our institution. Readability measures, including Flesch-Kincaid Grade Level (FKGL), and word count were calculated for the original and revised documents. The original and revised versions of the handouts were then scored in random order by two glaucoma specialists using the Suitability Assessment of Materials (SAM) instrument, a grading scale used to evaluate suitability of health information materials for patients. Paired *t* test was used to analyze changes in readability measures, word count, and SAM score between original and revised handouts. Finally, five glaucoma patients were interviewed to discuss the revised materials, and patient feedback was analyzed qualitatively.

**Results:**

Our literature search included 13 studies that evaluated a total of 950 educational materials. Among the mean FKGL readability scores reported in these studies, the median was 11 (representing an eleventh-grade reading level). At our institution, handouts’ readability averaged a tenth-grade reading level (FKGL = 10.0 ± 1.6), but revising the handouts improved their readability to a sixth-grade reading level (FKGL = 6.4 ± 1.2) (*p* < 0.001). Additionally, the mean SAM score of our institution’s handouts improved from 60 ± 7 % (adequate) for the original versions to 88 ± 4 % (superior) for the revised handouts (*p* < 0.001).

**Conclusions:**

Our systematic review of the literature reveals that ophthalmic patient education materials are consistently written at a level that is too high for many patients to understand. Our institution’s experience suggests that applying guidelines on writing easy-to-understand material can improve the readability and suitability of educational materials for patients with low health literacy.

## Background

Patient education materials are an important supplement to verbal communication with eye-care providers [1]. Informational handouts given at the end of a visit and patient-oriented webpages are popular forms of patient education material but often convey complex information at an advanced reading level [2]. High complexity and low readability make these materials difficult to comprehend for patients with a low level of health literacy, generally defined as the ability to read, understand, and act on health information [3]. Low health literacy is prevalent; the 2003 National Assessment of Adult Literacy found that 36 % of American adults have only basic or below basic levels of health literacy [4]. Furthermore, low health literacy is associated with billions of dollars in additional healthcare costs and poor health outcomes [5, 6].

Ophthalmology is no exception to the effect of low health literacy on health. For glaucoma patients, low health literacy is associated with worse vision-related quality of life [7], poor medication adherence [8], worsened visual field loss [9], and decreased understanding about glaucoma compared to patients with adequate health literacy levels [2, 9, 10]. Therefore, it is essential that ophthalmic patient education materials are written at accessible reading levels, both in print and online. The United States Department of Health and Human Services (USDHHS) recommends that patient education materials should be written at a sixth- to seventh-grade reading level (equivalent to years 7-8 in the United Kingdom (UK)) to make them accessible for patients with low health literacy.

The present study presents (1) a systematic review of the literature on readability of ophthalmic patient education materials and (2) evaluation and improvement of the readability and suitability of patient education materials at our own institution. Our academic ophthalmology practice previously developed patient education materials about glaucoma, which we sought to improve by applying a set of recommendations for writing easy-to-understand health material. We then utilized standardized scoring tools and patient interviews to evaluate how well our revisions improved our patient education materials.

## Methods

A systematic search for relevant studies on readability of ophthalmic patient education materials was conducted on the PubMed/MEDLINE database. The non-date-restricted search of English-language articles included the following key words: “readability ophthalmology,” “ophthalmology patient education,” and “ophthal* patient education materials.” To expand the search, references of included articles were examined for additional studies.

In addition to reviewing the literature, we also examined the readability and suitability of our own institution’s patient education materials about glaucoma—both before and after revising them using published guidelines. First, we collected evidence-based guidelines for writing easy-to-read patient education materials by compiling recommendations from three national organizations: “Creating and Using Patient-Friendly Written Materials” by the American Medical Association Foundation [11], “Simply Put: A Guide for Creating Easy-to-Understand Materials” by the Centers for Disease Control and Prevention [12], and “How to Write Easy-to-Read Health Materials” by the National Institutes of Health [13]. We used these guidelines to revise our patient education materials about glaucoma. Our focus was not to change the materials’ educational content, but instead to improve their readability, structure, and presentation for patients with low health literacy.

After all documents were revised, the original and revised handouts were scored in random order by two glaucoma specialists (KWM, JAR). Neither evaluator had a role in making the revisions, and neither had seen the revised documents before the masked scoring process. Both evaluators scored all handouts independently using the Suitability Assessment of Materials (SAM) instrument.

The SAM instrument is a widely used rating tool that systematically assesses the suitability of health information materials for a given patient population [14]. The SAM scoring tool has been validated and used to evaluate patient education materials for a number of diseases, including congestive heart failure and chronic kidney disease [15, 16]. SAM rates materials on factors that affect readability and comprehension, which makes it an ideal scoring tool when evaluating the suitability of patient education materials for patients who are low in health literacy. The SAM criteria are each given 0, 1, or 2 points based on adequacy of the handout to address each criterion, with 0 indicating a “not suitable” rating, 1 indicating an “adequate” rating, and 2 indicating a “superior” rating. The total SAM score for each handout is calculated as the sum of earned points divided by the number of possible points. The final SAM score reported as a percentage, with 70-100 % indicating superior material, 40-69 % adequate material, and 0-39 % unsuitable material.

In addition to SAM score, the word count and readability were assessed for each of the original and revised handouts. Readability scores were calculated as Flesch-Kincaid Grade Level (FKGL) and Flesch Reading Ease Score (FRES), which have been widely used in assessing patient education materials from various fields [17, 18], including ophthalmology [19]. FKGL indicates the academic grade level required to understand written material, determined by a formula that considers the number of words per sentence and the number of syllables per word [14]. For example, a FKGL score of 6.4 indicates that material is written at a sixth- to seventh-grade reading level (equivalent to years 7-8 in the UK). The FRES uses the same variables with an output between 0 and 100, with a higher score indicating that the material is more easily understandable.

Pearson correlation was calculated to determine inter-observer correlation between SAM scores assigned by the two evaluators. Paired *t* test was used for analysis of SAM scores, word count, FKGL, and FRES between original and revised patient education materials.

Finally, one-on-one interviews were conducted to receive input from glaucoma patients about the revised handouts. Our interview protocol was approved by the Duke University Institutional Review Board and adhered to the Declaration of Helsinki. Patients were recruited from our glaucoma service to review and to evaluate handouts on various topics with a study team member. Written informed consent was obtained from all participants. English-speaking adult patients age 18 years or older were eligible to participate if they had a diagnosis of glaucoma or glaucoma suspect, including ocular hypertension. Potential participants were excluded if they were unable to read or had vision worse than 20/70 in their better-seeing eye. All participants reviewed the “Top 10 for Glaucoma Patients” handout and selected a second or third topic of their choice from the handouts listed in Table 4. Subjects rated the documents with a validated eleven-question survey about the overall design quality of the handout, called the Consumer Information Rating Form (CIRF) [20], and they answered open-ended interview questions about the handouts (Table 7). Health literacy level was measured using the Rapid Estimate of Adult Literacy in Medicine – Short Form (REALM-SF) [21], and demographic information was obtained. Interview recordings were transcribed, and general themes were derived by iteratively coding responses and developing and applying an analytical framework, consistent with the framework method [22]. All three investigators coded themes independently and compared results.

## Results

### Overview

We first report the results of our literature review, which includes data from our study on evaluating and improving patient education materials at our institution. The results of our study are then presented in detail.

### Systematic review of the literature

Our search yielded 456 results between the three keyword searches. Duplicates were removed, and only studies evaluating patient education material in the field of ophthalmology were considered. In total, 12 studies were identified in our literature search, and addition of our study results makes for a total of 13 studies included in our review (Table 1).

**Table 1:** Characteristics of the 13 included studies

Study	Materials Evaluated	*n* Articles Evaluated	Medium	Evaluation Methods	Results	Conclusions
Barbosa and Martins (2007) [26]	Internet information about floaters and light flashes found by two search engines, MetaCrawler and MSN.	49	Online	FKGL and quality component scoring system	Mean FKGL = 9.9	“It is important for ophthalmologists not only to help to develop good-quality websites but also to direct their patients to sites that provide accurate information.”
Brown, et al. (2004) [25]	Cataract information leaflets from 12 ophthalmology departments in England	12	Print	SMOG, criteria for obtaining informed consent	Mean SMOG = 10	SMOG scores exceed recommended level of 5 or lower
Ebrahimzadeh, et al. (1997) [24]	Educational brochures from the AAO	22	Print	FKGL, FRES, GFI	32 % were at or below an 8th-grade reading level, 55 % between 8th- and 10th-grade levels, and 15 % were a 10th-grade reading level or higher	“With aid of computer programs, reading levels of materials can be analyzed and revised to reflect low health literacy”
Edmunds, et al. (2013) [19]	The “top 10 patient-oriented websites for 16 different ophthalmic diagnoses”	160	Online	FKGL, FRES, SMOG, GFI	Mean FKGL = 11.3	“[R]eadability scores were inferior to those recommended, irrespective of the measure used....we recommend the use of readability scoring when producing such resources in the future.”
Edmunds, et al. (2014) [27]	“[O]nline literature specifically for Graves’ disease and thyroid-associated ophthalmopathy” by Google search	50	Online	FKGL, FRES, SMOG, GFI	Mean FKGL = 11	“None of the web pages evaluated had readability scores in accordance with published guidelines....Screening of this online material, as well as subsequent revision, is crucial to increase future patient knowledge, satisfaction, and compliance.”
Hansberry, et al. (2014) [28]	Patient education material on AAO website	unspecified	Online	FKGL, FRES, SMOG, GFI, Coleman-Liau Index, the New Fog Count Formula, the New Dale-Chall Readability Formula, FORCAST formula, Raygor Readability Estimate, and the Fry Graph	Mean FKGL = 11.7	“[W]e believe revisions in line with the recommendations of the NIH may be warranted to improve patient comprehension”
Huang, et al. (2015) [29]	Websites from 7 ophthalmologic organizations: AAO, American Association of Ophthalmic Oncologists and Pathologists, AAPOS, AGS, ASCRS, ASOPRS, American Society of Retina Specialists, American Uveitis Society, Cornea Society, and NANOS	339	Online	FKGL, FRES, SMOG, GFI, Coleman-Liau Index, New Fog Count, New Dale-Chall Readability Formula score, FORCAST score, Raygor Readability Estimate Graph score, and Fry Readability Graph score.	Mean FKGL ranged from 10.4 to 12.6	“Online PEMs on major ophthalmologic association websites are written well above the recommended reading level. Consideration should be given to revision of these materials to allow greater comprehension among a wider audience”
John, et al. (2015) [30]	First 10 PEMs to appear in search on Google search for 10 pediatric ophthalmology conditions	100	Online	FKGL, FRES, SMOG, GFI, Coleman-Liau Index, New Dale-Chall, FORCAST Formula, Fry Graph, Raygor Reading Estimate, and the New Fog Count	Mean FKGL = 11.75	Only 12 % of articles were written below a 9th-grade level and only 3 % met recommended criteria.
Khurana, et al. (2003) [23]	Various ocular medication inserts	10 glaucoma medication inserts, 6 nonglaucoma inserts	Print	FKGL, SMOG	Mean FKGL = 12.9 (glaucoma inserts), Mean FKGL = 11.1 (nonglaucoma inserts)	All medications reviewed were written above an 8th-grade reading level
Martins and Morse (2005) [31]	Websites about retinopathy of prematurity found by two search engines, MetaCrawler and MSN.	40	Online	FKGL and quality component scoring system	Mean FKGL = 10.83	“In the majority of the sites (62.5 %) the ROP information was fair or poor.”
Muir and Lee [2] (2010)	Educational brochures from the AAO (Revised in 2008), NIH, NEI, AGS, the Glaucoma Research Foundation, and Prevent Blindness America	49	Print	FKGL	Mean FKGL = 8.3 (AAO), 9.7 (non-AAO)	“Unfortunately, there is still a dearth of written ophthalmic educational materials available from any agency for the least literate patients, precisely those who are at the greatest risk of blindness.”
Williams, et al.^a^	Duke Eye Center Glaucoma Guide (patient education material about various glaucoma topics written by Duke faculty)	12	Print	FKGL, FRES, SAM, word count	Mean FKGL = 10.0 (before revision); Mean FKGL = 6.4 (after revision)	Revisions of patient education materials using existing guidelines significantly improved their readability and suitability for a low-health-literacy population
Zaidi and Jones (2009) [32]	Websites that appear in the first 5 pages of a Google search for three terms related to blepharoplasty	101	Online	Objective quality of each website was measured using JAMA criteria: declaration of authorship, attribution of sources, disclosure of conflict of interests, and provision of date content was posted or updated (1 point for each, maximum 4 points total)	“Most sites scored low for quality—40 % scored zero for objective quality; 41 % scored just one point; 10 % scored two points; 6.5 % of sites scored three points; only 2.5 % of sites scored favourably on all four criteria.”	“This study identifies the poor quality of information on oculoplastic surgery, which is available to patients using the internet”

^a^The present study

*AAO* American Academy of Ophthalmology, *AAPOS* American Association for Pediatric Ophthalmology and Strabismus, *AGS* American Glaucoma Society, *ASCRS* American Society of Cataract and Refractive Surgery, *ASOPRS* American Society of Ophthalmic Plastic and Reconstructive Surgery, *FKGL* Flesch-Kincaid Grade Level, *FRES* Flesch Reading Ease Score, *GFI* Gunning Fog Index, *JAMA* Journal of the American Medical Association, *NANOS* North American Neuro-Ophthalmology Society, *NEI* National Eye Institute, *NIH* National Institutes of Health, *PEMs* Patient Education Materials, *SAM* Suitability Assessment of Materials, *SMOG* Simple Measure of Gobbledygook

Among the 13 studies, eight evaluated patient education material online and five in print, including one that evaluated ocular medication inserts [23]. Between all included studies, 950 articles were evaluated in total, although this figure almost certainly includes duplicates, as the same webpages were likely included in multiple studies. The included studies evaluated material such as educational brochures from ophthalmologic organizations [2, 24], information leaflets from English ophthalmology departments [25], educational material developed at an academic eye center, and webpages available to patients on the Internet [19, 26–32].

Various measures of readability and quality were used to evaluate educational material in the included studies. The most commonly reported metric was the Flesch-Kincaid Grade Level (FKGL), which ranged from 6.4 (the revised handouts in our study) to 12.9 (glaucoma medication inserts) [23]. Notably, the second-lowest reading grade level, 8.3, also comes from revised material (American Academy of Ophthalmology brochures revised in 2008) [2]. Among all reported mean FKGL scores, the median mean across all studies is 11, representing an eleventh-grade reading level (equivalent to year 12 in the UK).

Ebrahimzadeh, et al. [24] first evaluated the readability of ophthalmic patient education materials in 1997 and found that only 32 % of brochures published by the American Academy of Ophthalmology (AAO) were written at or below an eighth-grade reading level. Muir and Lee [2] later demonstrated that 38 AAO brochures, revised in 2008, improved significantly in readability since 1997, but many still fell short of the recommended sixth- to seventh-grade reading level; the average FKGL was 8.3 (i.e., eighth grade), with a range of 5.1 to 11.4. Muir and Lee also evaluated eleven patient education materials from other non-profit organizations, none of which was written at the recommended reading level (mean 9.7, range 8.4-12.0) [2]. Of all studies in this review, Khurana, et al. [23] reveal that medication inserts fare worst in readability; nonglaucoma medication inserts averaged an FKGL of 11.1, and glaucoma medication inserts scored 12.9, a readability at the university education level.

Although many patients use the Internet to learn about eye diseases [1], readability of online ophthalmic patient information does not fare better than print. Among the largest web-based studies, Edmunds, et al. [19] reviewed ten webpages for 16 different eye conditions in 2013. Of the 160 total websites from commercial and non-profit organizations, the mean FKGL was 11.3, with a range of 8.5 to 15.1. Not a single webpage adhered to the USDHHS guideline of a sixth- to seventh-grade reading level. In 2015, Huang, et al. [29] expanded upon this work by evaluating the readability of 339 online patient education materials from seven ophthalmologic association websites. Not a single document was written at or below the recommended sixth-grade reading level, and the authors conclude with a call for revising online patient education materials. Smaller studies of online material focused on topics like flashes and floaters [26], thyroid-associated ophthalmopathy [27], common pediatric ophthalmology conditions [30], and retinopathy of prematurity [31], and all concluded that the information available scores poorly in readability and requires revision (Table 1).

### The Duke experience: Improving suitability and readability of patient education materials

Twelve patient education handouts about glaucoma at our academic eye center were revised according to published guidelines (Table 2). Inter-observer correlation of SAM scores between the two evaluators was significant at a Pearson correlation of 0.73 (*p* < 0.01, *n* = 24). The revised handouts represent a significant improvement in scoring criteria and total SAM score compared to the original handouts (Table 3). Specifically, the mean (± standard deviation) SAM score improved from 60 ± 7 % (adequate) for the original versions (*n* = 12) to 88 ± 4 % (superior) for the revised handouts (*n* = 12) (*p* < 0.001). Criteria from all five graded areas improved upon revision (content, literacy demand, layout and type, learning stimulation and motivation, and cultural appropriateness) (Table 3). The SAM score for all 12 glaucoma topics improved upon revision (Table 4).

**Table 2:** Guidelines used for revising patient education materials

General Content
• Focus on 2-3 key concepts.
• Limit content to what patients really need to know.
• Use only words that are well known to individuals without medical training.
• Make certain content is appropriate for age and culture of the target audience.
• Identify action steps. State in beginning and repeat in the end of the document.
Text Construction
• Keep within a range of about a 6th to 8th grade reading level.
• Use one- to two-syllable words.
• Use short paragraphs.
• Use active voice.
• Use a clear topic sentence at the beginning of each paragraph. Follow the topic sentence with details and examples.
• Examples and stories may help engage readers.
• Use words like “you” instead of “the patient.”
• Structure the material logically, but include your most important points at the beginning of the document.
• You need to grab the reader’s attention at the beginning. People often do not read all the text and may miss your key point if you save the best for last.
• Some users prefer step-by-step instructions. Others may find concepts arranged from the general to the specific easier to understand.
• Use bulleted lists instead of blocks of text to make information more readable.
• Include specific actions the reader may or should take. Your document’s purpose should not be solely to inform but also to get the reader to take an action.
• Avoid abstract words in instructions for actions.
• Be consistent with terms.
• Emphasize the benefits of the desired behavior.
• Do not make assumptions about people who read at a low level. Don’t talk down to the reader. Maintain an adult perspective.
Visual Presentation
• Use colors that are appealing to your target audience.
• Use illustrations and photos with concise captions. Keep captions close to photos and illustrations.
• Avoid graphs and charts unless they actually help understanding. If you do use them, make sure they are simple and clear.
• Balance the use of text, graphics, and white space. Try for 40-50 % white space.
• Avoid using all capital letters. Upper and lower case are easier to read. To show emphasis, use bold, larger type size or different fonts.
• Avoid italics of more than a few words at a time.
• Make print large enough for your target audience. For most readers text the equivalent of Times New Roman 12 point is adequate. For seniors, consider using 14 point.
• Use easy-to-read fonts, such as Times New Roman, Arial, Tahoma and Helvetica.
• Use bolded headings and subheadings to separate and highlight document sections.
• When possible, use graphics or spell out fractions and percentages.
• Only justify the left margin. This means the left margin should be straight and the right margin should be “ragged.”
• Do not print text on top of shaded backgrounds, photos, or patterns.

**Table 3:** SAM criteria and SAM scores for original versus revised handouts

SAM Criteria		SAM Score		
		Original Handouts (*n* = 12)	Revised Handouts (*n* = 12)	*p* value
Content	(a) Purpose is evident	1.29	1.63	0.011
	(b) Content about behaviors	1.25	1.71	0.033
	(c) Scope is limited	1.88	1.92	0.341
	(d) Summary or review included	0.63	1.38	0.003
Literacy demand	(a) Reading grade level	0.33	1.25	<0.001
	(b) Writing style, active voice	1.13	1.88	0.001
	(c) Vocabulary	1.04	1.96	<0.001
	(d) Context is given first	1.42	1.71	0.271
	(e) Advance organizers	1.75	2.00	0.167
Layout and typography	(a) Layout factors	0.88	1.96	<0.001
	(b) Typography	1.63	2.00	0.026
	(c) Subheadings (“chunking”) used	1.33	2.00	0.003
Learning stimulation and motivation	(a) Interaction used	0.25	0.55	0.081
	(b) Behaviors are modeled and specific	1.58	1.83	0.082
	(c) Motivation--self-efficacy	1.55	1.92	0.015
Cultural appropriateness	(a) Match in logic, language, experience	1.25	1.96	0.001
	(b) Cultural image and examples	N/A	N/A	N/A
Total SAM Score (%) (mean ± SD):		60 ± 7	88 ± 4	<0.001

*SAM* Suitability Assessment of Materials, *SD* standard deviation

SAM criteria are graded 0, 1, or 2, and total SAM score is reported as a percentage of points earned out of total possible points

**Table 4:** SAM scores, word count, and readability level by handout topic

	SAM Score		Word Count		Flesch-Kincaid Grade Level		Flesch Reading Ease Score	
Handout Topic	Original	Revised	Original	Revised	Original	Revised	Original	Revised
Acute Glaucoma	51 %	91 %	251	212	8.6	5	60	75
Advice for Family Members	63 %	93 %	583	326	11.4	6.3	46.2	66.5
Chronic Angle Closure Glaucoma	54 %	83 %	358	223	10.8	4.6	53.4	79.1
Cataract Surgery Glaucoma Patients	55 %	89 %	844	632	9.5	7.3	55.8	63.7
Glaucoma Medications	63 %	88 %	833	550	10.4	6.5	52.8	70.1
Overview of the Glaucoma Team	50 %	84 %	689	559	12	8.2	42.2	59.1
What You Should Know Before Glaucoma Surgery	59 %	88 %	733	643	11.7	7.2	43.6	64.7
What You Need to Know After Glaucoma Surgery	70 %	89 %	775	507	9.3	4.8	58.2	76.4
What to Expect from Glaucoma Surgery	72 %	86 %	654	608	9.3	6.9	56	65.9
“Top Ten” for Glaucoma Patients	69 %	81 %	494	489	6.6	5.8	69.4	72.4
What to Expect on Your Visit	54 %	94 %	694	717	11.8	8.3	44.3	59.2
When to Call Your Eye Doctor	57 %	89 %	335	391	8.9	6.4	58.2	67
Average:	60 %	88 %	604	488	10	6.4	53	68
Standard Deviation:	7 %	4 %	201	166	1.6	1.2	8	6
Paired *t*-Test:	*p* < 0.001		*p* = 0.006		*p* < 0.001		*p* < 0.001	

*SAM* Suitability Assessment of Materials

In addition to suitability score, readability level also improved after revision of the original handouts. The average FKGL improved from 10.0 ± 1.6 to 6.4 ± 1.2 (*p* < 0.001), the mean FRES increased from 53 ± 8 to 68 ± 6 (*p* < 0.001), and the average word count decreased from 604 ± 201 to 488 ± 166 (*p* = 0.006) (Table 4).

The revised patient education materials were further evaluated by glaucoma patients. Following a regularly scheduled office visit, a total of five study subjects agreed to participate in a one-on-one interview to reflect on the quality of the handouts. Subjects ranged from 31 to 75 years of age, with a mean of 58 years. On the REALM-SF, four subjects scored at “ninth grade level or higher” and one scored at the “fourth-to-sixth grade” level. Demographic characteristics are summarized in Table 5.

**Table 5:** Demographic Characteristics of Interviewed Glaucoma Patients

Subject ID	Age	Gender	Race/Ethnicity	Eye Conditions	History of Eye Surgery	Highest Degree	First Language	REALM-SF Score
S1	47	Male	Latino/Chicano	Glaucoma, diabetic retinopathy	Yes	Some high school	Spanish	4th-6th grade level
S2	70	Male	White/European-American	Glaucoma, cataracts	No	College degree	English	≥ 9th grade level
S3	31	Male	White/European-American	Ocular hypertension	No	College degree	English	≥ 9th grade level
S4	66	Female	White/European-American	Glaucoma, cataracts	No	Not reported	English	≥ 9th grade level
S5	75	Female	White/European-American	Glaucoma, cataracts	Yes	Some college	English	≥ 9th grade level

*REALM-SF* Rapid Estimate of Adult Literacy in Medicine—Short Form

Before the interview, subjects completed Consumer Information Rating Form (CIRF) evaluations for “Top Ten List for Glaucoma” and another topic of their choosing. The CIRF scales range from 1 to 5, with higher scores indicating better quality. Four different educational topics were selected and evaluated, with all five subjects rating “Top Ten List for Glaucoma.” The highest scoring areas of this scale were organization and finding the handout helpful, but almost all CIRF items rated above a four on the five-point scale. (Table 6).

**Table 6:** Patient evaluation of revised handouts

CIRF Item	Score Mean (SD)
How easy or hard is the handout to read?	3.9 (0.9)
How easy or hard is the handout to understand?	4.0 (0.5)
How easy or hard is the handout to remember?	3.7 (0.7)
How easy or hard is the handout to locate information?	3.9 (0.6)
How easy or hard is the handout to keep for future reference?	4.1 (0.7)
How organized is the handout?	4.6 (0.7)
How attractive is the handout?	4.2 (0.8)
How is the print size?	4.3 (0.7)
How is the tone of the handout?	4.1 (0.8)
How helpful is the handout?	4.7 (0.5)
How is the spacing between lines?	4.4 (0.7)

*CIRF* Consumer Information Rating Form

Items rated 1 through 5, with higher scores indicating better quality

Structured interviews were conducted using open-ended questions outlined in Table 7, and interview transcripts revealed themes of patient preferences. In particular, subjects emphasized using concrete language, providing practical information, and having a simple format. A positive tone and emphasis that the provider is available to help were other characteristics that stood out to interviewed subjects. Although subjects were mixed about keeping the handout for reference or reading it only once, all generally appreciated having the key points highlighted. For some handouts, a picture of the eye or graphic of drop instillation were suggested, but images were not perceived as necessary for all handouts. Lastly, while brevity is essential, our subjects also desired a picture of the road ahead to glean what to expect during the course of their experience with glaucoma. Table 8 pairs themes with supporting quotations.

**Table 7:** Structured interview questions

General Evaluation of the Handouts
• Is this handout helpful?
• What catches your eye?
• Who do you think this handout is for?
• What do you think about the handouts?
• What do you like about the handouts?
• What would you want to change about the handouts?
• Is there information that you don’t need?
• Is there anything that you don’t like?
• Which handout is the best? Why?
• Which handout is the worst? Why?
Comprehension
• Tell me in your own words what this is trying to say.
Readability
• Do you see any words that you think some people might have a hard time understanding?
• Is anything confusing?
Past Experiences with Patient Education Handouts
• Do you get handouts like these from your doctor?
• Do you like educational handouts?
• Do you usually read them?
• Do you save them to reference later?
• What information do you like to see in handouts?
• What don’t you like about patient education handouts?
Graphics
• What would you like to see in a picture?
• Would a picture be helpful?
• What are your thoughts on having a video to go along with the handouts?

**Table 8:** Themes Derived from Patient Interviews

Theme	Supporting Quotations
Emphasize that we are available to help	S1: “If you forgot something and need some help, you know who to call about the drops.”
	S3: “I like that at the top it says in bold ‘the short answer is that if you’re worried, call.’ I think that leaves one with the idea that you’re not going to be a nightmare patient if you’re calling. I don’t think anyone wants to feel like that, like they’re the patient that’s calling too much or being a little anxious for no reason.”
Be concrete	S2: “You miss two or three appointments, you might lose your eyes. So missing appointments is pretty vague, but the repercussions are very costly.”
	S2: “Doing them both at the same time [cataract surgery and glaucoma surgery] will lower your eye pressure. My question is, what does that do for you? If you lower the eye pressure, I assume that’s good. If it’s high it’s not good.”
	S2: “There are a couple of sentences that were sort of confusing to me. Like this one: ‘Make sure you understand how much vision loss you have [from glaucoma].’ How are you supposed to know that?”
	S3: “I think it’s good that it [the post-operative handout] says, ‘if you have pain that’s not improved by Tylenol.’ You’re going to have pain [after surgery], but if it’s not going away with a light pain reliever, then you should call.”
	S5: “I liked the idea that it was stressing that by missing any drops…how that can be detrimental.”
Give practical information	[Interviewer: *What stood out most to you about this one?*]
	S1: The reminders. Like setting your iPhone to remember [when to take your drops].
	S3: “It’s got good practical information....About remembering to take a refill with you when you’re traveling, that’s a good reminder. Having people write a schedule is a practical piece of advice, and, you know, it’s good that it stresses the importance of the consistency of the appointments.”
Keep it simple	S3: “It keeps it pretty simple. I think someone’s family member could read this and be like, okay, I’m on the same page with my loved one here.”
	S5: “Absolutely, [I] prefer one page [for the length of the handout].”
Highlight key points	S2: “Some people that are real busy would look at the highlights, skim through it. See what seems important....You could highlight a few things like exercise or whatever that’s important, that type of thing. It’s the things I look at whenever I read these types of things, the things that I focus in at. People don’t read more than 2 or 3 pages at a time.”
	S3: “The emergency number is at the bottom. I would maybe move that to the top.”
Give a picture of the road ahead	S2: “It seems like when I get involved [have the need for cataract surgery], this would be a good starting point for me, to answer questions for me.”
	S3: “I don’t have it [glaucoma] yet, you know, I guess I’m a candidate because of the pressure, but I think with that in mind its got good information.”
	S3: “If you’re new to this as I’m going to be when I have the drops, it’s good for someone who’s younger like me who has a risk, I don’t think it’s bad to mention to have them somewhere convenient, by your bathroom, by your bedside, in the kitchen, with other medications.”
	S3: “It’s saying you’ve got to keep an eye on this stuff [post-operative complications] for a long time and be careful about observing it.”
Maintain a positive tone	S3: “It’s caring, sensitive, helpful, and it’s positive. You stay on top of this and everything should be, you know, that will be the best for you. So it’s encouraging in the sense of, just stay on top of this condition, and, you’ll be in a better place as a patient.”
	S3: “It encourages you to educate yourself, and once you’ve met with one of you all, you kind of educate yourself about the risk factors; in my case, the eye pressure. So you just kind of leave knowing that you just have to take your drops and just kind of stay on top of it.”
Provide source for more information	S3: “I like the encouragement to learn about it [referring to the website to go to for more information on glaucoma], I think that lends to the positive nature about the handout, you know, go learn more about it, educate yourself. I guess doctors say an educated patient is an empowered patient.”
	S4: “I like this website. Because I do try to read everything I can find on glaucoma.”
Illustrate	S1: Would like a picture of “what glaucoma looks like in the eye.”
	S2: “Definitely pictures are better. I’m not too graphic, but color would be a good if it’s a picture of the eye. Help people understand better.”
	S3: Desires “an image about the optic nerve and where that is. Like an eye diagram.”
	S5: “Perhaps there could be a little bit more of the demonstration or more of a verbal detailed aspect of actually putting the drop in your eye because that was traumatic for me.”

## Discussion

Ophthalmic patient education materials are written at a difficult readability level, both in print and online. We conducted a systematic review of 13 studies that measured readability or quality of ophthalmic patient education materials, and we found that the median Flesch-Kincaid Grade Level (FKGL) across all studies represents an eleventh-grade reading level, which far exceeds the level that many patients can understand. Various measures of readability and quality were used across the studies in this review, but all papers called for improvements in the material available for ophthalmic patients. Despite a universal call for improvement, no included study assessed methods for how to improve readability of patient education material. Drawing from other literature, we revised our patient handouts at our academic eye center using guidelines for writing easy-to-understand material (Table 2), and these changes significantly improved their readability and suitability for a low-health-literacy population. Additionally, we solicited feedback from glaucoma patients, and individual interview sessions reflected published guidelines, including providing practical information, being concrete, and highlighting the key points. This process has demonstrated that patient feedback is a valuable tool to ensure proper implementation of these recommendations.

Previous literature has examined effective recommendations for improving patient education materials. These studies outline steps for writing [33–35] and revising [36–39] health education material, and the recommendations reflect the collated list in Table 2, with an emphasis on maintaining an accessible readability level. Putting one of these recommendations into practice, Sheppard, et al. [36] improved readability level of patient education websites in orthopedics by shortening sentences to no more than 15 words, as recommended by NIH guidelines. This simple intervention improved the readability of eight articles by an average of 1.41 grade levels. Recommendations from various sources can be used to improve readability and suitability levels of patient education materials in ophthalmology and other specialties.

Despite a history of poor readability scores, ophthalmic patient education materials may be improving for patients with low health literacy. The AAO recently released updated versions of its patient education brochures in 2014, which are written at an eighth-grade level or lower and feature improved font and format for patients with low vision and patients with a low health literacy level [40].

Although these improvements should be lauded, many important patient education topics are not commercially available as brochures. Critical information in ophthalmology, such as pre-operative instructions and post-operative expectations, is not readily available for purchase, and specific content will vary depending on the individual ophthalmic practice or surgeon. As such, many ophthalmology clinics must develop at least some patient education material on their own. The guidelines presented in Table 2 may be a helpful reference for other groups to revise the suitability and readability of their ophthalmic patient education material with attention to low health literacy. In fact, even patients with a high level of health literacy prefer and more easily understand simplified language in written healthcare materials [10, 41, 42]. Lastly, with the overwhelming volume of medical information available to patients, clinicians and institutions share a responsibility in the “arc of health literacy” for population health to provide critical take-home messages that patients can easily understand [43, 44].

Our study has several limitations. First, we restricted our literature search to studies on ophthalmic patient education materials in the English language, which excludes a body of work on readability published in other fields and limits generalizability to non-English-language material. Second, readability is just one component of measuring suitability of patient education material, and we suggest that future studies include comprehensive suitability scores, such as SAM [14], to include factors such as layout, content, and learning stimulation. Third, in our study, we evaluated patient education material about glaucoma from a single academic ophthalmology practice, which may not be generalizable to other organizations. Additionally, feedback from glaucoma patients came from only five subjects, and just one had health literacy below the ninth-grade level. Finally, we did not measure patient knowledge of glaucoma or monitor health behavior after reading the handouts, as these metrics are outside the scope of this study.

## Conclusions

Our systematic review of research on ophthalmic patient education materials showed that materials are consistently written at a readability level that is poorly suited for patients with low health literacy. Fortunately, patient educational materials can be improved. By revising our institution’s educational handouts using guidelines on writing easy-to-understand material (Table 2), we significantly improved the documents’ suitability for patients with low health literacy; the average reading level decreased from the tenth-grade level to the sixth-grade level after revision. Additionally, feedback from glaucoma patients demonstrated positive evaluation of the handouts, and open-ended patient interviews provided further insight for areas of improvement. A similar systematic approach of applying the guidelines we collated in Table 2 may improve the suitability, readability, and patient evaluation of other ophthalmic educational materials.
